# Suitable endometrial thickness on embryo transfer day may reduce ectopic pregnancy rate and improve clinical pregnancy rate

**DOI:** 10.1186/s12884-023-05837-6

**Published:** 2023-07-15

**Authors:** Shiming Wang, Lin Qi, Yaping Liu, Hao Shi, Xiaoli Chen, Ningning Wang, Yingchun Su

**Affiliations:** Reproductive Medical Center, Henan Province Key Laboratory of Reproduction and Genetics, The First Affiliated Hospital of Zhengzhou University, No. 1 East Jianshe Road, Erqi District, Zhengzhou, China

**Keywords:** Endometrial thickness, Ectopic pregnancy, Clinical pregnancy, Fresh embryo transfer, IVF/ICSI

## Abstract

**Background:**

This retrospective study aimed to investigate the most suitable endometrial thickness (EMT) on the day of embryo transfer that could reduce ectopic pregnancy rate (EPR) and improve clinical pregnancy rate (CPR) in fresh embryo transfer patients with early follicular phase long-acting regimen.

**Methods:**

A total of 11,738 IVF/ICSI cycles, comprising 4,489 non-clinical pregnancies, 7,121 intrauterine pregnancies, and 128 ectopic pregnancy cycles after fresh embryo transfer, recorded between September 2017 and December 2020. Clinical pregnancy (CP) and ectopic pregnancy (EP) were the primary outcomes. Multivariate logistic regression was used to calculate the adjusted odds ratio (aOR) and 95% confidence interval (CI) for EP and CP. Patients were divided into three groups based on the EMT (6–10 mm, 11–15 mm, and 16–20 mm). CPR and EPR per millimeter of EMT were drawn into a line chart, and three groups were analyzed by Chi-square test.

**Results:**

After controlling for potential confounders, EMT had a significant effect on CP (aOR = 1.07; 95% CI, 1.05–1.08; *P* = 0.00) and EP (aOR = 0.88; 95% CI, 0.82–0.94; *P* = 0.00). With the increase of EMT, CPR increased and EPR decreased. Pearson correlation coefficients were *r* = 0.708 (*P* = 0.00) and *r* =-0.558 (*P* = 0.03), respectively. Significant differenceswere detected in the CPRs and EPRs (all *P* = 0.00). The CPR in the 6–10 mm group (54.88%) was significantly lower than that in the 11–15 mm group (64.23%) and the 16–20 mm group (64.40%) (*P* = 0.00). The EPR in the 6–10 mm group (2.72%) was significantly higher than that in the other two groups (1.60% and 0.97%, *P* = 0.00). The difference in CPR and EPR between the 11–15 mm group and the 16–20 mm group was not statistically significant, which indicated that EMT ≥ 11 mm simultaneously reduced the EPR and increased the CPR.

**Conclusions:**

EMT was inversely proportional to EPR and directly proportional to CPR in fresh embryo transfer cycles. The EMT ≥ 11 mm on the day of embryo transfer could simultaneously achieve lower EPR and higher CPR. Accordingly, more attention should be given to the EMT of women who underwent ART treatment.

**Supplementary Information:**

The online version contains supplementary material available at 10.1186/s12884-023-05837-6.

## Background

With the rapid development of assisted reproductive technology (ART), the clinical pregnancy rates (CPR) have substantially improved; however, the incidence of ectopic pregnancy (EP) is increasing. The ectopic pregnancy in IVF/ICSI cycle, which was reported to be 2.1%-8.6% [1, 2], is more frequent than 1–2% [3] observed in the natural pregnancy group, thus suggesting that ART independently increases the EP risk [4]. Furthermore, 95% of EP occurs in the fallopian tubes, resulting in the injury and removal of reproductive organs such as fallopian tubes. In some severe cases, it may even lead to early maternal death [5], thus causing a heavy psychological and economic burden. Multiple factors may be responsible for the high rate of EP during the IVF/ICSI, EP history, tubal factors, pelvic lesions, smoking, ART procedures, and similar [6–9].

Endometrial thickness (EMT) is a frequently monitored indicator in the IVF/ICSI process and an indirect indicator of endometrial receptivity. Several studies have focused on the EMT in terms of the relationship between the EMT and pregnancy outcome, placenta previa risk and birth weight, etc. Existing studies have shown that the EMT is positively correlated with the CPR in the IVF/ICSI [10–13]. On the contrary, some believe that the EMT is not associated with CPR [13, 14] and is a poor predictor of CPR [15, 16]. Yuan et al*.* [11] reported that the EMT was a statistically significant predictor of CP and EP. Rombauts et al. demonstrated that thin endometrium is an independent risk factor for EP, and patients with EMT < 9 mm have four times the EP risk of > 12 mm [17]. Another research also confirmed that EMT > 12 mm can reduce the risk of EP [18]. In contrast, a meta-analysis concluded that the EMT did not affect on the EP outcome [12].

The relationship between EMT, CP, and EP is controversial and inconclusive. In addition, there were no clear guidelines for recommendations on the EMT for reducing the EPR and increasing the CPR. Therefore, further discussion and research are needed. Our study focused on exploring the factor(s), which could simultaneously improve CPR and reduce EPR in fresh embryo transfer patients with early follicular phase long-acting regimens. First, we utilized a large sample size to define the risk factor(s) in EP and CP. Next, we identified EMT as the intrinsic factor that may be predictive in obtaining better pregnancy outcomes. Ultimately, we determined which EMT range can attain lower EPR and higher CPR.

## Methods

### Study design

**Fig. 1:**
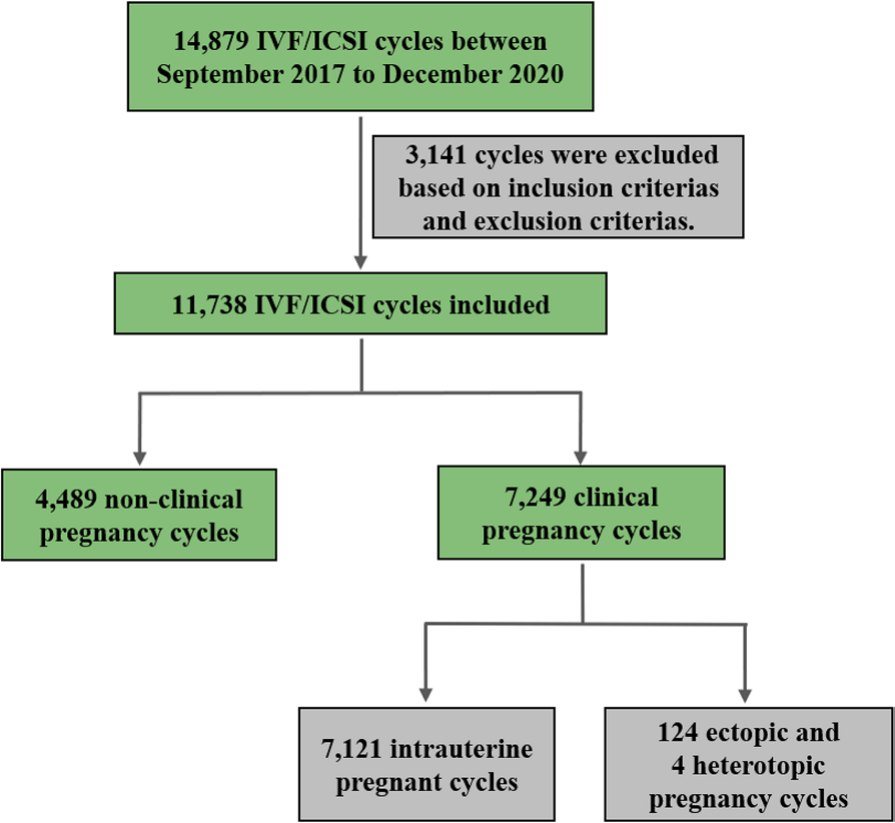
Patients’ selection flowchart

This retrospective study was based on 14,879 IVF/ICSI fresh cycles recorded at the Reproductive Medicine Center of the First Affiliated Hospital of Zhengzhou University between September 2017 and December 2020. Patients with early follicular phase long-acting regimens were included. The exclusion criteria were as follows: embryo transfer except on the D3 or D5; patients with uterine malformation or endometriosis; genetic factors infertility. After screening for exclusion criteria, 11,738 IVF/ICSI fresh cycles were included, comprising 4,489 non-clinical pregnancies, 7,121 intrauterine pregnancies, and 124 ectopic and 4 heterotopic pregnancies cycles (1.77%), as shown in the patients' selection flow chart (Fig. 1).

This retrospective study was approved by the Ethics Committee of the First Affiliated Hospital of Zhengzhou University. (reference number: 2022-KY-0340–002).

### Definition of clinical outcomes

The primary outcome measures were clinical pregnancy (CP) and ectopic pregnancy (EP). Biochemical pregnancy referred to pregnancy confirmed only by positive detection of hCG in serum or urine [19], determined at 14 or 18 days after embryo transplantation in our center. The CP was defined as pregnancy confirmed by ultrasound showing one or more pregnancy sacs [19]. Patients with biochemical pregnancy were confirmed by b-ultrasound reexamination at 35 days after an embryo transfer in our center. CPs were divided into intrauterine (IUP) and ectopic (EP) pregnancies. The IUP referred to the presence of the sac inside the uterus, and EP referred to the presence of the sac outside the uterus. Heterotopic pregnancy referred to the presence of a pregnancy sac, both intrauterine and extrauterine, also classified as the EP. The CPR was defined as the number of clinical pregnancies per 100 embryo transfer cycles, while the EPR was defined as the number of EP cycles per 100 clinical pregnancy cycles after embryo transfer. The EMT was measured on the midsagittal plane of the uterine body on the day of embryo transfer to measure the maximum thickness of the endometrium-muscularum junction from one interface to the other.

The spontaneous cessation of an intrauterine pregnancy before the completion of 22 weeks of gestation was known as spontaneous abortion. Missed miscarriage was defined as spontaneous loss of a clinical pregnancy before 22 weeks of gestational age, in which the embryo(s) or fetus(es) was/were nonviable and was/were not spontaneously absorbed or expelled from the uterus. Induced abortion referred to intentional loss of an intrauterine pregnancy, through intervention by medical, surgical or unspecified means. Preterm birth was defined as a birth that occured after 22 weeks and before 37 consecutive weeks of gestation.

### ART protocols and embryo assessment

Our patients were fresh embryo transfer patients with the early follicular phase long-acting regimen. Patients were injected with 3.75 mg of long-acting gonadotropin-releasing hormone agonist (GnRH-a, Diphereline, Ipsen, France) on day 2 of menstruation. Patients meeting reduced criteria were initiated with gonadotropin (Gn), usually recombinant follicle-stimulating hormone (r-FSH, Pouliquen, Merck Sharp & Dohme, USA) or human menopausal gonadotropins (HMG, Menopur, Hui Ling, Switzerland) to stimulate follicle growth within 30 to 40 days after injection. The down-regulated criteria were FSH < 5 mIU/mL, LH < 5 mIU/mL, E2 < 50 pg/ mL and EMT ≤ 5 mm. When ≥ 2 follicles reached a diameter of ≥ 18 mm or more than 2/3 of follicles reached ≥ 16 mm, ovulation was triggered by Aizer (250 µg, Merck Serono, Italy) or hCG (2,000 IU, Lizhu Medicine). Transvaginal ultrasound-guided oocyte retrieval was conducted approximately 37 h later. Depending on the patient's condition and embryo development, one or two D3/D5 embryo(s) were selected for transfer. Luteal support was initiated on the day of oocyte retrieval with daily transvaginal progesterone (Xenotong, Merck Serono, Switzerland) and oral progesterone (30 mg/d, Dupbaston, Abbott, Holland).

Day 3 embryos are graded employing Peter's criteria in our center based on the number, size, average degree, refractive index, and blastomere fragmentation [20]. Top-quality embryos were those classified as Grade I and II. On day 5, blastocysts were morphologically evaluated and scored utilizing Gardner's standard for blastocyst stage, inner cell mass (ICM) and trophectoderm (TE) [21]. The final choice to transfer fresh embryos was based on ultrasound results on the morning of day 3 or day 5.

### Statistical analysis and analyzed variables

Based on previously published articles and clinical experience, the following factors were analyzed in the present study: female age; female body mass index (BMI); basal follicle-stimulating hormone (FSH); infertility factor types; EMT on the day of embryo transfer; fertilization method (IVF or ICSI); the developmental days of transplanted embryos (D3 or D5); the number of embryos transferred (1 or 2); cycle number (1 or ≥ 2); and laboratory indicators (egg count; MII rate; 2PN rate; high-quality embryo rate; blastocyst formation rate).

Data were analyzed by SPSS25.0 software. The quantitative data were recorded as mean ± standard deviation (SD). The continuous variables with normal distribution were compared using Student’s t-test, while those with non-normal distribution were compared using the Wilcoxon rank-sum test. Qualitative data were recorded as frequency and percentage (%), and frequencies were compared using the Chi-square test or Fisher's exact test. We included all possible variables and included baseline comparisons of laboratory data that have not been addressed by previous studies. Data were analyzed with the multivariate logistic regression to calculate the adjusted odds ratio (aOR) and 95% confidence interval (CI) for CP and EP to control potential confounders and analyze the risk factors of the CP and EP. The CPR and EPR per millimeter of EMT were drawn into a line chart, and three groups were analyzed by Chi-square test. *P* < 0.05 was considered statistically significant.

## Results

### Predictive factors associated with CP and EP

The basic characteristics of all patients and cycles are shown in Table 1. The outcome variables were CP (Table 1) and EP (Table 2) separately. There were significant differences in female age, fallopian tube factors, polycystic ovary syndrome (PCOS), ovarian dysfunction, endometrial thickness, embryo transfer stage, the number of transplanted embryos, cycle number, and all laboratory data between the non-CP group and CP group (all *P* < 0.05). In contrast, significant differences were only found in tubal factors, EMT, and fertilization method between IUP and EP groups (all *P* < 0.05). Patients with tubal factors, thinner endometrium, or using IVF had a higher risk of EP, while many factors had no significant effect on EP (*P* > 0.05), including age, BMI, basal FSH, ovarian dysfunction, male factors, embryo transfer stage (D3 or D5), embryo transfer number, cycle number, and laboratory data.

**Table 1:** Basic characteristics with CP

	**non-CP**	**CP**	***P* value**
**Total**	4,489	7,249	
**Age (y)**	32.81 ± 5.41	30.39 ± 4.21	**0.00**
**Body mass index (BMI) (kg/m**^**2**^**)**	23.13 ± 3.19	23.08 ± 3.22	0.45
**Basal FSH (IU/L)**	7.07 ± 2.47	7.60 ± 75.20	0.64
**Infertility factors**			
Tubal factor	2,371 (52.82%)	3,531 (48.71%)	**0.00**
Polycystic ovary syndrome (PCOS)	296 (6.59%)	845 (11.66%)	**0.00**
Ovarian dysfunction	283 (6.30%)	209 (2.88%)	**0.00**
Male factor	331 (7.37%)	631 (8.70%)	**0.01**
Unexplained and others	1,208 (26.91%)	2,033 (28.05%)	0.18
**Endometrial thickness (mm)**	12.04 ± 2.85	12.52 ± 2.59	**0.00**
**Fertilization method**			0.95
IVF	3,447 (76.79)	5,563 (76.74%)	
ICSI	1,042 (23.21%)	1,686 (23.26%)	
**Stage of embryo transferred**			**0.00**
Day 3 embryo	3,396 (75.65%)	5,260 (72.56%)	
Day 5 embryo	1,093 (24.35%)	1,989 (27.44%)	
**The number of embryos transferred**			**0.00**
1	2,024 (45.09%)	2,557 (35.27%)	
2	2,465 (54.91%)	4,692 (64.73%)	
**The number of cycles**			**0.00**
**1**	3,870 (86.21%)	6,540 (90.22%)	
** ≥ 2**	619 (13.79%)	709 (9.78%)	
**laboratory indicators**			
Egg count	12.10 ± 5.95	13.34 ± 5.61	**0.00**
MII rate	0.82 ± 0.16	0.83 ± 0.15	**0.00**
2PN rate	0.66 ± 0.22	0.68 ± 0.18	**0.00**
High-quality embryo rate	0.67 ± 0.28	0.72 ± 0.24	**0.00**
Blastocyst formation rate	0.45 ± 0.34	0.53 ± 0.33	**0.00**

*FSH* Follicle-stimulating hormone, *IVF* in vitro fertilization, *ICSI* Intracytoplasmic sperm injection. Bold: *p* < 0.05. *P* < 0.05 was considered to be statistically significant

**Table 2:** Basic characteristics with EP

	**IUP**	**EP**	***P* value**
**Total**	7,121	128	
**Age (y)**	30.39 ± 4.21	30.34 ± 4.12	0.91
**Body mass index (BMI) (kg/m**^**2**^**)**	23.08 ± 3.21	23.20 ± 3.39	0.68
**Basal FSH (IU/L)**	7.61 ± 75.88	7.10 ± 2.64	0.94
**E**_**2**_** on hCG day (pg/mL)**	2876.79 ± 1375.70	2844.90 ± 1696.07	0.85
**P on the day before ET (ng/mL)**	98.05 ± 34.53	92.39 ± 34.82	0.13
**Infertility factors**			
Tubal factor	3,449 (48.43%)	82 (64.06%)	**0.00**
Polycystic ovary syndrome (PCOS)	837 (11.75%)	8 (6.25%)	0.07
Ovarian dysfunction	208 (2.92%)	1 (0.78%)	0.28
Male factor	625 (8.78%)	6 (4.69%)	0.14
Unexplained and others	2,002 (28.11%)	31 (24.22%)	0.38
**Endometrial thickness (mm)**	12.54 ± 2.59	11.68 ± 2.66	**0.00**
**Fertilization method**			**0.01**
IVF	5,452 (76.56%)	111 (86.72%)	
ICSI	1,669 (23.44%)	17 (13.28%)	
**Stage of embryo transferred**			0.19
day 3 embryo	5,160 (72.46%)	100 (78.13%)	
day 5 embryo	1,961 (27.54%)	28 (21.87%)	
**The number of embryos transferred**			0.15
1	2,520 (35.39%)	37 (28.91%)	
2	4,601 (64.61%)	91 (71.09%)	
**The number of cycles**			1.00
**1**	6,425 (90.23%)	115 (89.84%)	
** ≥ 2**	696 (9.77%)	13 (10.16%)	
**laboratory indicators**			
Egg count	13.34 ± 5.60	13.13 ± 6.43	0.71
MII rate	0.83 ± 0.15	0.83 ± 0.15	0.81
2PN rate	0.68 ± 0.18	0.65 ± 0.19	0.09
High-quality embryo rate	0.72 ± 0.24	0.71 ± 0.25	0.69
Blastocyst formation rate	0.53 ± 0.33	0.49 ± 0.33	0.11

*FSH* Follicle-stimulating hormone, *IVF* in vitro fertilization, *ICSI* Intracytoplasmic sperm injection. Bold: *p* < 0.05. *P* < 0.05 was considered to be statistically significant

In order to investigate the influencing factors of CP and EP, the multivariate logistic regression analysis was performed. All variables with *P* < 0.1 in the univariate analysis were included in the multivariate regression analysis (Tables 3 and 4). The results confirmed that the older age led to lower clinical pregnancy rate (aOR = 0.92; 95% CI, 0.91–0.93; *P* = 0.00). Patients with PCOS increased the risk of clinical pregnancy by 51% (aOR = 1.51; 95% CI, 1.30–1.77; *P* = 0.00). The probability of CP was reduced by 40% in patients with ovarian dysfunction (aOR = 0.60; 95% CI, 0.46–0.80; *P* = 0.00). The D5 embryo transfer was associated with a 52% increase in CPR compared with D3 (aOR = 0.66; 95% CI, 0.56–0.78; *P* = 0.00). Compared with single embryo transfer, double embryo transfer increased the CPR by about 1.4 times (aOR = 2.38; 95% CI, 2.06–2.75; *P* = 0.00). A high rate of 2PN (aOR = 1.50; 95% CI, 1.14–1.99; *P* = 0.00), high quality embryo rate (aOR = 1.75; 95% CI, 1.45–2.11; *P* = 0.00), blastocyst formation rate (aOR = 1.62; 95% CI, 1.41–1.87; *P* = 0.00) all promoted CP. Tubal factors resulted as risk factors for EP, increasing the risk of EP by 49% (aOR = 1.49; 95% CI, 1.01–2.18; *P* = 0.04). After controlling for potential confounding factors, EMT had a significant effect on the CP (aOR = 1.07; 95% CI, 1.05–1.08; *P* = 0.00) and the EP (aOR = 0.88; 95% CI, 0.82–0.94; *P* = 0.00). It can be concluded that the thicker intima, the higher the probability of CP, and the lower EP risk.

**Table 3:** Multivariate logistic regression analysis with CP

	**aOR**	**95% CI**	***P* value**
Age (y)	0.92	0.91–0.93	**0.00**
Tubal factor	0.95	0.86–1.04	0.25
PCOS	1.51	1.30–1.77	**0.00**
Ovarian dysfunction	0.60	0.46–0.80	**0.00**
Male factor	1.03	0.87–1.22	0.76
Endometrial thickness (mm)	1.07	1.05–1.08	**0.00**
Stage of embryo transferred	1.52	1.28–1.80	**0.00**
The number of embryos transferred	2.38	2.06–2.75	**0.00**
The number of cycles	0.96	0.84–1.11	0.60
Egg count	1.01	1.00–1.02	0.07
MII rate	1.22	0.87–1.71	0.26
2PN rate	1.50	1.14–1.99	**0.00**
High-quality embryo rate	1.75	1.45–2.11	**0.00**
Blastocyst formation rate	1.62	1.41–1.87	**0.00**

Bold: *p* < 0.05. *P* < 0.05 was considered to be statistically significant. *PCOS* Polycystic ovary syndrome

**Table 4:** Multivariate logistic regression analysis with EP

	**aOR**	**95% CI**	***P* value**
Tubal factor	1.48	1.01–2.18	**0.04**
PCOS	0.52	0.25–1.07	0.08
Endometrial thickness (mm)	0.88	0.82–0.94	**0.00**
Fertilization method	0.61	0.36–1.04	0.07
2PN rate	0.57	0.21–1.51	0.26

Bold: *p* < 0.05. *P* < 0.05 was considered to be statistically significant. *PCOS *Polycystic ovary syndrome

### The distribution of EMT

**Fig. 2:**
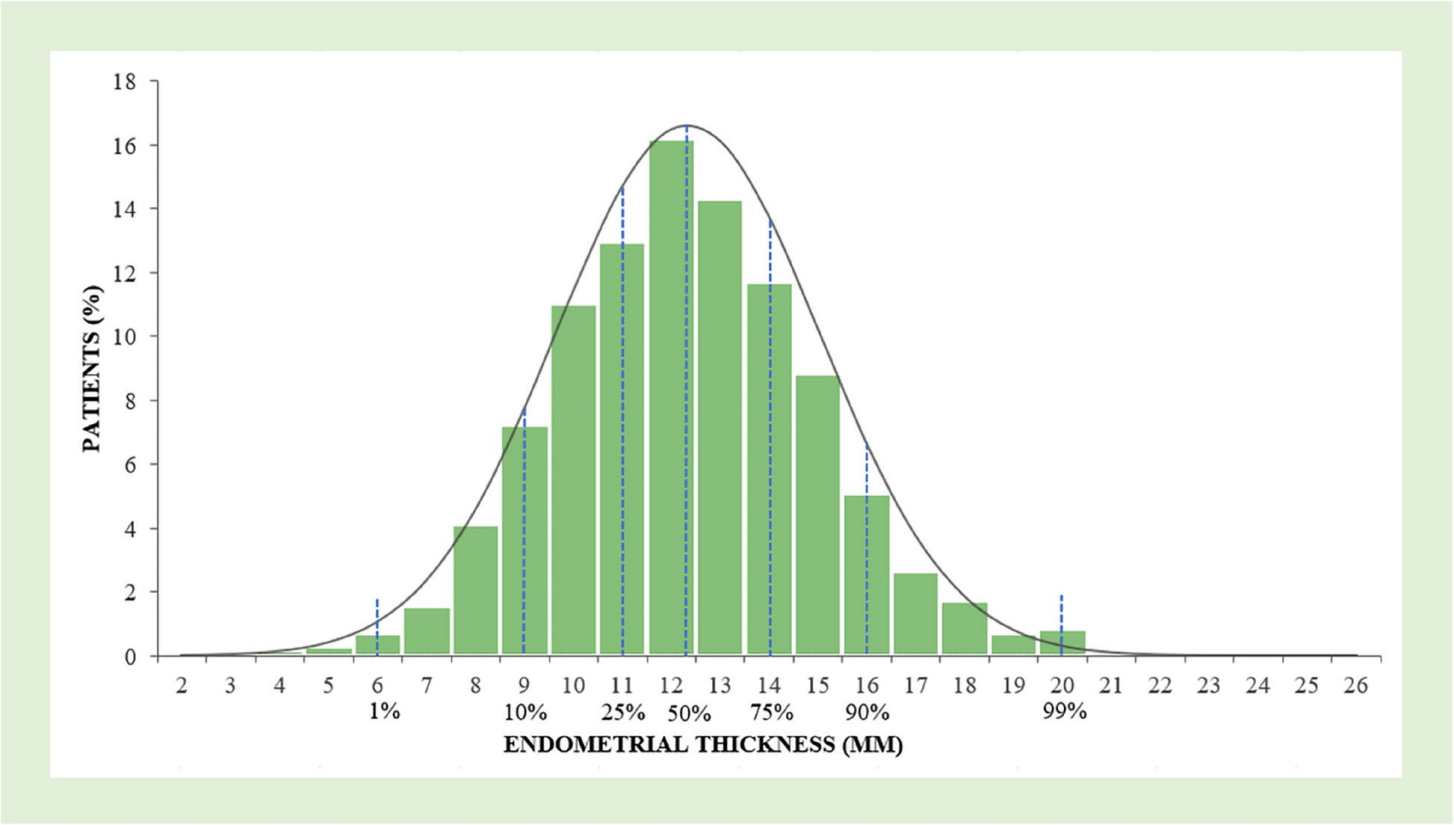
Distribution of patients by endometrial thickness on the day of embryo transfer

The distribution of EMT on the day of embryo transfer is shown in detail in Fig. 2. Mean (± SD) and median EMT were 12.34 (± 2.70) and 12.00, respectively. P1 = 6 mm, P10 = 9 mm, P25 = 11 mm, P50 = 12 mm, P75 = 14 mm, P90 = 16 mm, P99 = 20 mm. Also, 98% patients had EMT between 6–20 mm.

### CPR and EPR by per mm EMT and EMT groups

**Fig. 3:**
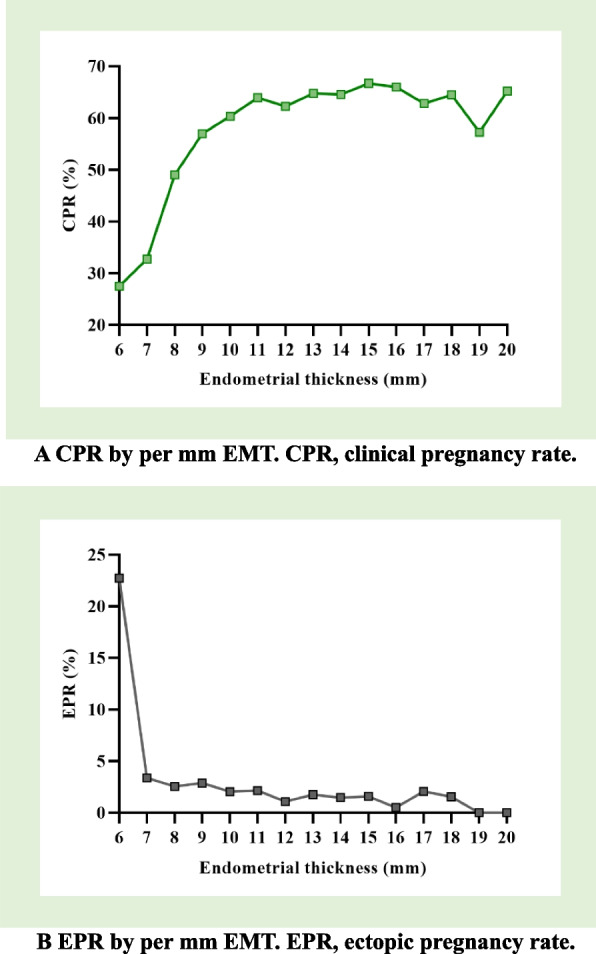
**A** CPR by per mm EMT. CPR, clinical pregnancy rate. **B** EPR by per mm EMT. EPR, ectopic pregnancy rate

The line charts of CPR (Fig. 3A) and EPR (Fig. 3B) per mm of EMT within the range of 6–20 mm had followed certain rules. With the increase of EMT, CPR increased and EPR decreased. The Pearson’s coefficient showed a significant association (*P* < 0.05) of EMT with CPR (*r* = 0.708 (high correlation, *P* = 0.003) and EPR (*r* =-0.558, medium correlation, *P* = 0.031). The linear regression models were CPR = 0.019 × EMT + 0.327 (*P* = 0.003) and EPR = -0.007 × EMT + 0.12 (*P* = 0.031). The CPR gradually increased at 6–10 mm, while it reached a platform area at ≥ 11 mm and gradually stabilized at 60% -70%, but then slightly declined at 16–19 mm. The EPR gradually declined at 6–10 mm, while it was roughly stable at 1%-2% when EMT ≥ 11 mm, although some values were outside the range. Additionally, line charts of live birth rate and early miscarriage rate per mm remained stable at ≥ 11 mm as shown in Figure S1.

**Fig. 4:**
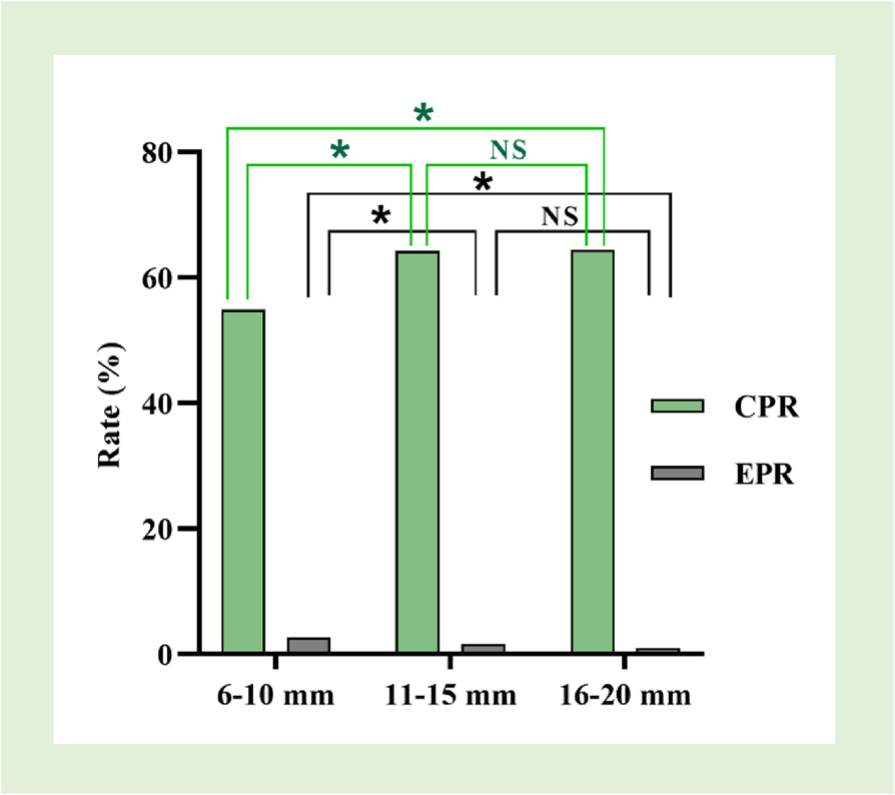
Comparisons of CPR and EPR by EMT groups. *P < 0.01. NS = not significant. CPR, clinical pregnancy rate; EPR, ectopic pregnancy rate

The comparison of CPR and EPR by EMT groups is described in Fig. 4. Significant differences were observed between CPR and EPR groups (*P* = 0.00). The CPR in the 6–10 mm group (54.88%) was significantly lower than that in the 11-15 mm group (64.23%) and the 16–20 mm group (64.40%) (*P* = 0.00), while EPR in the 6-10 mm group (2.72%) was significantly higher than that in the other two groups (1.60% and 0.97%, *P* = 0.00). Notably, the differences in CPR and EPR between the 11–15 mm group and the 16—20 mm group were not statistically significant, which indicated that EMT ≥ 11 mm simultaneously tended toward higher CPR and lower EPR.

## Discussion

In this large retrospective study, we analyzed 11,738 IVF/ICSI cycles aiming to identify the influencing factors of CPR and EPR in the IVF/ICSI so as to better define appropriate EMT on the day of embryo transfer to achieve better pregnancy outcomes. Our results confirmed that compared to EMT < 11 mm, the CPR was higher, and the EPR was lower when EMT ≥ 11 mm on the day of embryo transfer. In fact, after adjusting for all possible confounding factors, the impact of EMT on the day of ET persisted.

Our findings indicated that older age, thin endometrium, and ovarian dysfunction were the hindering factors for CP, while D5 embryo transfer, PCOS, double embryo transfer, high 2PN rate, high-quality embryo rate and blastocyst formation rate all promoted CP. Numerous studies have demonstrated that age is inversely correlated with CPR after IVF/ICSI [22]. The older the patients are, the worse the ovarian function, the worse the quality and quantity of oocytes and embryos, the thinner the endometrium, and the greater the obstacle to successful CP [14]. D5 embryo transfer has been identified as a definite CP promotion factor [23], corroborating that blastocyst formation rate is directly proportional to the CPR. Our results revealed that a high-quality embryo rate which represents the embryo quality was positively correlated with CPR, as shown by a previous study [24]. It was reported that the CPR and live birth rate of double embryo transfer was much higher than that of single embryo transfer [25]; nonetheless, opposite findings were also reported [26]. However, as double embryo transfer fortifies the risk of premature birth, low birth weight, cerebral palsy, and similar, elective single-embryo transfer (eSET) was advocated as the most effective mean to avoid multiple gestations in IVF cycles [27, 28].

The findings of this paper yield a puzzling result that PCOS could promote clinical pregnancy. Despite the fact that PCOS patients are at a greater risk of infertility due to ovulation abnormalities, they have an advantage in ovarian reserve [29]. The largest retrospective investigation on PCOS revealed that individuals aged 20–44 had considerably greater mean number of oocytes retrieved, clinical pregnancy and live-birth rates than the control group [30]. This has also been supported by previous investigations [31, 32]. Increased oocytes retrieved paves the way for more highly qualified embryos. The present study's results that PCOS encourages clinical pregnancy can also be explained by those aforementioned findings. Additionally, we compared PCOS patients with non-PCOS patients to confirm this and discovered that PCOS patients were younger in age (29.28 ± 3.78 vs 31.53 ± 4.90, *P* = 0.00), had higher number of oocytes retrieved (15.57 ± 5.73 vs 12.57 ± 5.70, *P* = 0.00), more high-quality embryos (6.96 ± 3.97 vs 5.46 ± 3.58, *P* = 0.00) and higher CPR (74.1% vs 60.4%, *P* = 0.00). These findings line up with the research mentioned above.

It was found that EMT and tubal infertility were independent risk factors for EP, while fertilization method, embryo development days (D3 or D5), and a number of transferred embryos had a futile effect on the EP. Several studies have concluded that D5 and D3 fresh embryo transfer made no difference in EP risk [18, 23]. BMI and PCOS were not significantly correlated with EP in the present study, which was consistent with previous studies [33, 34]. Tubal infertility, which was identified as an important risk factor for the EP during IVF-ET [35]. Due to controlled ovarian hyperstimulation (COH) during the IVF/ICSI, high estrogen status may influence uterine receptivity and tubal status leading to higher EP rates [36]. Moreover, the relatively higher E-cadherin expression, a kind of cell adhesion molecule mediating embryo implantation at the fallopian tube in the IVF/ICSI patients, is another possible reason [37].

Multiple studies have reported that thin endometrium is associated with lower CPR and higher EPR [10, 11, 17, 18, 38, 39], which is also consistent with our findings. The reasons why thin endometrium leads to higher EPR and lower CPR are complex and undetermined. One is the effect of oxygen tension, where implanted embryos are closer to the spiral arteries in the basal endometrium layer due to thin or absent uterine functional layer, which results in exposure to higher oxygen concentration that restrains their growth [40]. Furthermore, abnormal uterine peristalsis may increase the risk of the embryo being dislodged from its original transfer position, which may increase the likelihood of EP [41, 42].

It was found that the EMT ≥ 11 mm on the embryo transfer day could simultaneously achieve lower EPR and higher CPR in the present study, supported by a number of other observations. Fang et al*.* [39] demonstrated that EP risk was reduced by about 50% when EMT was > 10 mm. The cut-off value of EMT as a possible predictor of IUP was 10 mm(AUC = 0.69) [43]. Rombauts and colleagues [17] found that patients with an EMT > 12 mm had a threefold lower risk of developing EP compared with patients with an EMT < 9 mm. Similarly, patients with EMT > 12 mm had a lower EP risk [18]. In the present study, patients with a 4 mm EMT achieved CP, while there are previous reports of CP with an EMT of 4 mm and 4.8 mm [44]. Patients with thin endometrium could realize pregnancy, but the EP risk is relatively high. Nevertheless, there is still a high CPR when EMT < 11 mm. A large EMT (> 14 mm) had no adverse effect on the CPR, which is in line with previous studies [10]. Still, it was reported that the CPR significantly decreased when EMT > 14 mm [45], and patients with EMT > 12 mm had a fourfold higher risk of placenta previa than those with EMT < 9 mm [46]. Therefore, it is imperative that patients pursue an appropriate EMT, and the findings in the present study counsel that patients with an EMT of 11 mm or greater have a higher chance of CP and a lower risk of EP.

The timing of EMT measurements has been described variously in the literature of EMT and IVF-ET outcome studies, and the ideal moment of measurement representing endometrial receptivity remains contentious. Less research has chosen the ET day, while the majority of studies have picked the day of hCG or oocyte retrieval for endometrial assessment. An increasing ET day EMT has been exhibited in earlier research to favor pregnancy outcome in both fresh and FET cycles [47]. According to a recent meta-analysis, ET day EMT is a somewhat unreliable prospective indicator of pregnancy outcomes [15]. As opposed to oocyte retrieval day and ET day, Dechaud et al.'s research [48] observed that endometrial assessment on the day of the hCG injection was more pertinent to the clinical pregnancy outcome. Undeniably, the endometrium on the ET day is the one with which the transferred embryo came into direct contact. The EMT on the ET day of patients with fresh embryo transfer is routinely measured in our center. Few studies have explored the relationship between ET day endometrium and ectopic pregnancy, to which we added that the relationship between ET day EMT and EP is consistent with other studies.

### Strengths and limitations

We believe that EMT on the ET day is when the endometrial environment is directly contacted by the transferred embryo, which can reduce the influence of confounding factors such as possible endometrial changes, thus making comparative analysis more reliable. Additionally, this study has strict standards of arrangement, a large sample size, and a wide range of EMT. All included patients were fresh embryo transfer patients with the early follicular phase long-acting regimen, which reinforced the statistical reliability. A single-center study reduced variability between centers. Unlike other studies, this study focused on the suitable EMT that simultaneously resulted in a lower EPR and a higher CPR.

One limitation of the present study is related to its observational nature that is associated with some inevitable shortcomings and does not take all the confounding factors such as race, transfer fluid volume, and transferring depth etc. [4] into account. Smoking is a well-known confounding factor that was not recorded in the data, but the number of patients who smoked was so small that it should not have affected the results. In addition, although there are uniform standards for single-center trained sonographers, there were some differences between individuals. Besides, there may be selection bias considering the population was from a single center. Consequently, future multi-center studies are needed to further confirm reported results.

## Conclusion

EMT resulted inversely proportional to EPR and directly proportional to CPR in fresh embryo transfer cycles. The EMT ≥ 11 mm on the embryo transfer day could simultaneously achieve lower EPR and higher CPR. Future studies are needed to evaluate the predictive validity of EMT and to validate the applicability to other regimes of IVF/ICSI.

## Supplementary Information

**Additional file 1:**  **Figure S1. **LBR and EMR by per mm EMT. LBR, live birth rate; EMR, early miscarriage rate.

## Data Availability

The datasets used and/or analysed during the current study are available from the corresponding author on reasonable request.
