# Supplementary figures and images for: Suitable endometrial thickness on embryo transfer day may reduce ectopic pregnancy rate and improve clinical pregnancy rate
Source: BMC Pregnancy Childbirth. 2023 Jul 15;23:517. doi: 10.1186/s12884-023-05837-6 (PMC10349401; doi:10.1186/s12884-023-05837-6)

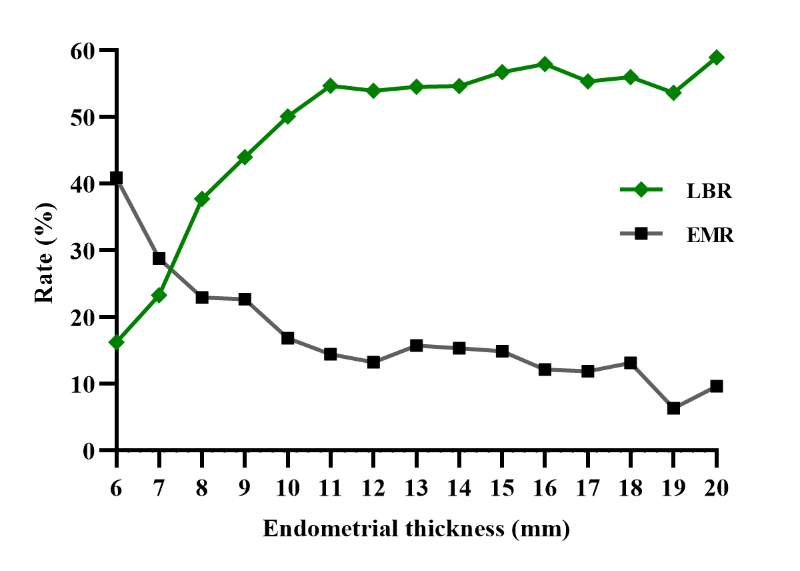


**Figure S1 LBR and EMR by per mm EMT. LBR, live birth rate; EMR, early miscarriage rate.**

Supplement: Supplementary file 1 — Additional file 1: Figure S1. LBR and EMR by per mm EMT. LBR, live birth rate; EMR, early miscarriage rate. [file 12884_2023_5837_MOESM1_ESM.docx]
